# Parallel and Sequential Sequences of Taste Detection and Discrimination in Humans

**DOI:** 10.1523/ENEURO.0010-19.2019

**Published:** 2019-01-25

**Authors:** Rosalind SE Carney

## Abstract

**Highlighted Research Paper:** As Soon as You Taste It: Evidence for Sequential and Parallel Processing of Gustatory Information by, Raphael Wallroth and Kathrin Ohla

Rapid perception of gustatory information enables humans to detect and discriminate between substances that may be nutritious or noxious, enabling immediate expulsion of food from the mouth that may be harmful. In particular, bitter and sour tastes can be a sign that food may be poisonous or rotting, respectively. The different basic taste categories, sweet, salty, sour, bitter, and umami, are first distinguished by taste receptor cells that are predominantly located on the tongue. Taste categories vary with respect to signaling receptor subtype, signaling speed, and hedonics. For example, salty and sour taste signaling occurs via ion channels and has faster taste detection response times and faster cortical response latencies than sweet and bitter tasting substances which signal via G protein coupled-receptors (Pfaffmann, 1955; Yamamoto and Kawamura, 1981; Kuznicki and Turner, 1986; Kobayakawa et al., 1999; Crouzet et al., 2015). In terms of palatability, salty and sour tastes can be iso-hedonic if the concentration is adjusted well, whereas bitter and sweet tastes vary hedonically at almost any concentration. In humans, taste detection occurs within 200 ms, whereas taste discrimination takes 100–200 ms longer (Lester and Halpern, 1979; Yamamoto and Kawamura, 1981). However, it was not known whether the cognitive processes underlying taste discrimination occur sequentially or in tandem with the early stages of sensory encoding that mediate taste detection. In their *eNeuro* publication, Wallroth and Ohla, 2018 addressed this question by comparing electrophysiological recordings of neural activity to behavioral response times in humans undergoing taste detection and discrimination tasks.

Twenty participants aged 18–34 years completed two forced-choice tasks while ongoing 64-channel electroencephalogram (EEG) recording was performed. Figure 1 shows a schematic illustration of the experimental design. A gustometer delivered three fine mist 70-µl sprays of tastant solution (salty, bitter, sour, or sweet) or water to the anterior surface of the tongue. The tastants were similar in taste intensity and tastant solutions and water rinses were maintained at 38°C to minimize thermal sensations; no tastant solutions or rinses were swallowed by the participants.

**Figure 1.**
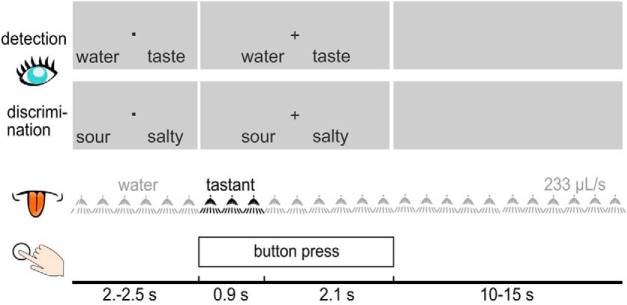
Schematic overview of the experimental design of the taste detection and discrimination tasks. At the beginning of the trial, a fixation dot appeared in between the two possible response options. When the tastant was being presented, the fixation dot was replaced by fixation cross to prompt the subjects to press a button indicating their response. A rinsing period preceded the next trial. (Fig. 1 in Wallroth and Ohla, 2018.)

At the beginning of each taste detection trial, participants saw a fixation dot in between two possible response options, water or tastant (Fig. 1). At the time the tastant was presented, the fixation dot was replaced by a fixation cross, prompting the participants to respond as soon as possible via button press to indicate whether they detected water or a tastant. At the beginning of each taste discrimination trial, participants saw a fixation dot in between two possible response options, either sour/salty or bitter/sweet (Fig. 1). At the time the tastant was presented, the fixation dot was replaced by a fixation cross, prompting the participants to respond as soon as possible via button press to indicate which tastant they detected.

Wallroth and Ohla performed multivariate pattern analysis on the recordings to determine the time point at which taste detection or discrimination occurred within individual trials. In essence, a machine-learning algorithm was trained to detect signal variations, for example, a tastant-related activity change relative to the signal elicited by water in the detection task. For the discrimination task, the signal variation reflected the different signal responses that each tastant elicited.

The results showed that participants were able to detect the presence of salty and sour tastants faster than they were able to discriminate between these tastes categories. The neural onset of detection occurred 100 ms before the onset of discrimination, thereby mirroring the behavioral findings. This observation is in agreement with previous studies that described a lag in the timing of discrimination of salty and sour taste categories compared to detection (Yamamoto and Kawamura, 1981; Halpern, 1986; Kuznicki and Turner, 1986). Wallroth and Ohla also found that sweet and bitter tastants took longer to be detected than salty and sour tastants. However, discrimination of bitter and sweet tastants occurred instantly as soon as the taste was detected. Again, the neural onsets mirrored the behavioral findings.

These observations confirm that the cognitive processes underlying behavioral responses to taste are closely associated with the early stages of taste-related neural activity. However, whether taste detection and discrimination occur in mostly parallel (sweet and bitter) or mostly sequential (salty and sour) sequences due to differences in signaling receptor type or hedonics is unknown. These findings represent an advance in the field of taste perception that animal models cannot provide and give insight into the patterns of neural activity that can mediate the acceptance or avoidance of different taste categories.
